# Adaptation of the socioecological model to address disparities in engagement of Black men in prostate cancer genetic testing

**DOI:** 10.1186/s12889-024-20008-8

**Published:** 2024-09-18

**Authors:** Amy E. Leader, Timothy R. Rebbeck, William K. Oh, Alpa V. Patel, Eric P. Winer, LeeAnn O. Bailey, Leonard G. Gomella, Crystal Y. Lumpkins, Isla P. Garraway, Lisa B. Aiello, Monica L. Baskin, Heather H. Cheng, Kathleen A. Cooney, Amanda Ganzak, Daniel J. George, Susan Halabi, Feighanne Hathaway, Claire Healy, Joseph W. Kim, Michael S. Leapman, Stacy Loeb, Kara N. Maxwell, Christopher McNair, Todd M. Morgan, Breanne Prindeville, Howard R. Soule, Whitney L. Steward, Sakinah C. Suttiratana, Mary-Ellen Taplin, Kosj Yamoah, Thierry Fortune, Kris Bennett, Joshua Blanding-Godbolt, Laura Gross, Veda N. Giri

**Affiliations:** 1 Department of Medical Oncology, Sidney Kimmel Cancer Center, Thomas Jefferson University, Philadelphia, PA USA; 2 Department of Epidemiology, Harvard TH Chan School of Public Health and Dana-Farber Cancer Institute, Boston, MA USA; 3 Department of Internal Medicine, Mount Sinai Hospital, New York, NY USA; 4 Department of Population Science, American Cancer Society, Atlanta, GA USA; 5 Department of Medicine, Yale Cancer Center and Yale School of Medicine, 333 Cedar Street, WWW214A, New Haven, CT 06520 USA; 6 National Cancer Institute/Center to Reduce Cancer Health Disparities, Rockville, MD USA; 7 Department of Communication, Population Sciences Division, Huntsman Cancer Institute, University of Utah, Salt Lake City, UT USA; 8 Department of Urology, David Geffen School of Medicine, University of California, Los Angeles, CA USA; 9 VA Greater Los Angeles Healthcare System, Los Angeles, CA USA; 10 Corporal Michael J. Crescenz VA Medical Center, Philadelphia, PA USA; 11 Department of Medicine, UPMC Hillman Cancer Center, University of Pittsburgh School of Medicine, Pittsburgh, PA USA; 12 Department of Medicine, Fred Hutchinson Cancer Center, University of Washington, Seattle, WA USA; 13 Department of Medicine, Duke University School of Medicine and Duke Cancer Institute, Durham, NC USA; 14 Cancer Genetics and Prevention Program, Yale New Haven Hospital, New Haven, CT USA; 15 Department of Biostatistics & Bioinformatics, Duke University School of Medicine, Durham, NC USA; 16 Department of Medicine, High-Risk and Advanced Prostate Cancer Clinic, University of Chicago Medicine, University of Chicago, Chicago, IL USA; 17 Department of Urology, Yale School of Medicine, New Haven, CT USA; 18 Department of Urology and Population Health, New York University and Manhattan Veterans Affairs, New York, NY USA; 19 Department of Medicine-Hematology/Oncology, Perelman School of Medicine, University of Pennsylvania, Philadelphia, PA USA; 20 Department of Urology, Rogel Cancer Center, University of Michigan, Ann Arbor, MI USA; 21 Neaman Center for Personalized Medicine, NorthShore University Health System, Evanston, IL USA; 22 Prostate Cancer Foundation, Santa Monica, CA USA; 23 Department of Medicine, Dana-Farber Cancer Institute, Boston, MA USA; 24 Departmetnt of Radiation Oncology, H Lee Moffitt Cancer Center and Research Institute, Tampa, FL USA; 25 Movember, Santa Monica, CA USA; 26 Department of Medicine, Yale University and Yale Cancer Center, New Haven, CT USA

**Keywords:** Prostate cancer, Genetic testing, Disparities, Health equity

## Abstract

**Background:**

Black men consistently have higher rates of prostate cancer (PCA)- related mortality. Advances in PCA treatment, screening, and hereditary cancer assessment center around germline testing (GT). Of concern is the significant under-engagement of Black males in PCA GT, limiting the benefit of precision therapy and tailored cancer screening despite longstanding awareness of these disparities. To address these critical disparities, the Socioecological Model (SEM) was employed to develop comprehensive recommendations to overcome barriers and implement equitable strategies to engage Black males in PCA GT.

**Methods:**

Clinical/research experts, national organization leaders, and community stakeholders spanning multiple regions in US and Africa participated in developing a framework for equity in PCA GT grounded in the SEM. A novel mixed-methods approach was employed to generate key areas to be addressed and informed statements for consensus consideration utilizing the modified Delphi model. Statements achieving strong consensus (> =75% agreement) were included in final equity frameworks addressing clinical/community engagement and research engagement.

**Results:**

All societal levels of the SEM (interpersonal, institutional, community, and policy/advocacy) must deliver information about PCA GT to Black males that address benefits/limitations, clinical impact, hereditary cancer implications, with acknowledgment of mistrust (mean scores [MS] 4.57-5.00). Interpersonal strategies for information delivery included engagement of family/friends/peers/Black role models to improve education/awareness and overcome mistrust (MS 4.65-5.00). Institutional strategies included diversifying clinical, research, and educational programs and integrating community liaisons into healthcare institutions (MS 4.57-5.00). Community strategies included partnerships with healthcare institutions and visibility of healthcare providers/researchers at community events (MS 4.65–4.91). Policy/advocacy included improving partnerships between advocacy and healthcare/community organizations while protecting patient benefits (MS 4.57-5.00). Media strategies were endorsed for the first time at every level (MS 4.56-5.00).

**Conclusion:**

The SEM-based equity frameworks proposed provide the first multidisciplinary strategies dedicated to increase engagement of Black males in PCA GT, which are critical to reduce disparities in PCA-mortality through informing tailored screening, targeted therapy, and cascade testing in families.

**Supplementary Information:**

The online version contains supplementary material available at 10.1186/s12889-024-20008-8.

## Introduction

 Prostate cancer (PCA) consistently remains among the highest in both cancer incidence and mortality affecting US males. Black individuals with a prostate (herein referred to as “males”) have 1.4-fold greater PCA incidence and 1.7-fold greater risk of death from PCA [1]. A revolution in the treatment of metastatic, castration-resistant PCA (mCRPC) has heralded the era of precision medicine. Treatment for mCRPC now centrally includes on germline (genetic) testing (GT) to inform options for PARP inhibitors upfront or upon progression for men who carry pathogenic/likely pathogenic variants (mutations) in host of genes including *BRCA2*,* BRCA1*,* ATM*, DNA mismatch repair genes, *CHEK2*, and *PALB2*, among other genes [2]. Furthermore, PCA screening guidelines advocate for starting screening at a younger age (age 40 years) compared to the general population for males who carry mutations in *BRCA2*,* BRCA1*,* ATM*,* CHEK2*,* PALB2*,* HOXB13*,* MLH1*,* MSH2*,* MSH6*,* PMS2*,* EPCAM*, and *TP53* due to higher risk for PCA and aggressive disease for some of these genes [3]. Multiple genes associated with PCA are linked with hereditary cancer syndromes which predispose to multiple cancer types for individuals and their blood relatives [4]. Given the expansion of clinical indications, current NCCN guidelines recommend germline testing for all males with metastatic PCA, high-risk or node-positive disease, and strong family cancer history [2, 5]. 

However, the lower engagement of Black males in PCA GT is a major concern for widening of disparities and worse clinical outcomes. Most PCA genetic studies among men eligible for GT have had 5–15% participation of Black males [4], which matches clinical experience of low patient representation in most clinical genetics programs. Furthermore, precision medicine studies have historically had < 10% participation of Black males [2]. These disparities may be due to multiple factors including barriers to accessing healthcare, lack of awareness of the benefit of GT and precision medicine, and/or mistrust of GT and the healthcare system [6–10], which hinder engagement in PCA GT and limit the benefit of precision medicine and tailored cancer screening. While there is longstanding awareness of these factors impacting cancer disparities, there remains consistent under-engagement of eligible Black males in PCA GT [2, 4]. As advances in oncology increasingly center on genetics, it is critical to develop implementation frameworks that address engagement of diverse populations in GT.

Here, we adapted the Socioecological Model (SEM) [11] to drive the development of two novel multilevel societal frameworks with recommendations to reach and engage Black males for PCA GT who meet national guidelines to reduce PCA disparities in the precision medicine era.

## Methods

### Use and adaptation of Socioecological Model (SEM)

**Fig. 1:**
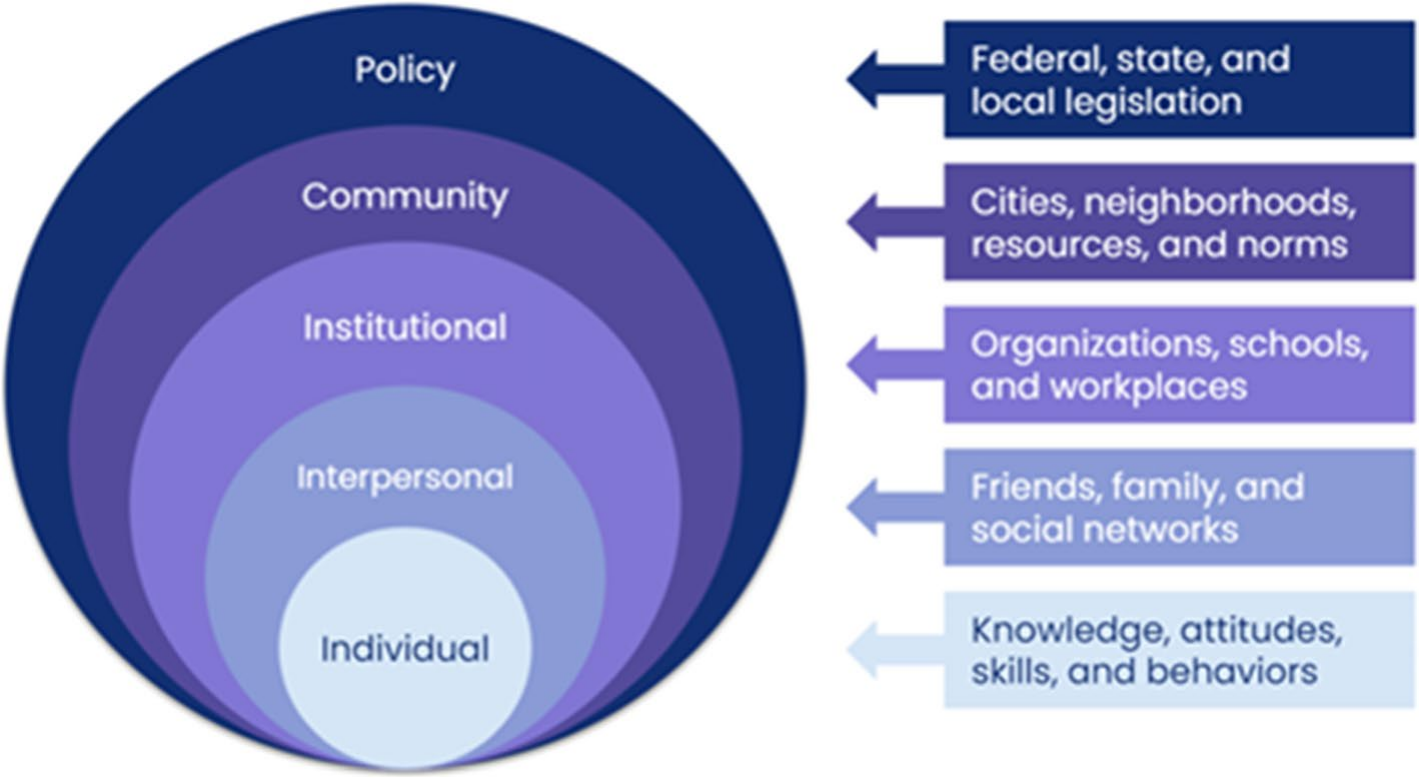
Socioecological model for understanding influences on human behavior and health. Model used to guide qualitative and quantitative methods for the project based on the content of Sallis et al. 2008 [11].

The methods for equity framework development were grounded in the SEM, a conceptual model for understanding the influences on human behavior and health [11]. The model considers the dynamic interplays between individuals and their environments while acknowledging that behavior is shaped through multilevel factors including the individual/intrapersonal, interpersonal, institutional, community, and policy levels (Fig. 1) [11]. Overarching questions to address were: (1) What strategies should be employed to increase engagement of Black males in PCA GT? (2) What strategies should be implemented to increase engagement in PCA genetics research?

#### Participants in framework development

A stakeholder conference was convened that included 23 participants spanning medical oncology, radiation oncology, urology, genetics/genomics, basic science research, population science, clinical research, genetic counseling, community members, health communication, and policy/advocacy. Multiple national organizations were represented including the Prostate Cancer Foundation, Center to Reduce Cancer Health Disparities of the National Cancer Institute, American Cancer Society, American Society of Clinical Oncology, ZERO Prostate Cancer, and Research Advocacy Network. Participants were from across the United States and the African continent. Table 1 displays participant demographics.

**Table 1:** Characteristics of Roundtable Conference voting participants (*n* = 23 participants)

Characteristics	*N* (%)^a^
Specialty	
• Medical Oncology	8 (35%)
• Genetics/Genomics/Genetic Counseling	8 (35%)
• Population Science	6 (26%)
• Urology	3 (13%)
• Clinical Research	3 (13%)
• Community Member	3 (13%)
• Radiation Oncology	1 (4%)
• Basic Science Research	1 (4%)
• Advocacy	1 (4%)
• Other: Community Engaged Research	1 (4%)
• Other: Community Outreach and Engagement	1 (4%)
**Geographic Region**	
United States:	
• National reach^b^	16 (70%)
• Northeast US	10 (43%)
• Midwest US	4 (17%)
• Southeast US	3 (13%)
• Southwest US	1 (4%)
• Northwest US	1 (4%)
• Other: Intermountain West	1 (4%)
African continent	2 (9%)
**Work Setting**	
Academic medical center/hospital	21 (91%)
Advocacy organization	4 (17%)
Veterans Affairs	3 (13%)
Community Site	3 (13%)
Research Laboratory	2 (9%)

^a^Participants could choose multiple responses. Therefore, percentages do not total 100%

^b^Given as an option to capture participants from national organizations, which go beyond regional reach

### Evidence review

Evidence summaries included: overview of PCA genetics and disparities [2–4, 12–22]; community engagement for GT; [23, 24] addressing disparities and enhancing equity in oncology care; [25–27] models for enhancing community engagement in oncology and genetics; [28–30] patient perspectives regarding PCA, GT, and impact on families; [31–33] clinical impact of PCA GT for metastatic disease, screening, and genetic counseling and risk communication [2–4, 12–22, 34–37]; peer-based approaches to enhance community engagement; [38–40] considerations of GT in the Veterans Health Administration; [41, 42] GT from the patient and physician perspective; [43, 44] and global perspective on PCA GT [45, 46].

### Thematic analysis of key elements of SEM

After evidence review, participants were divided into two discussion groups focused on levels of the SEM and impact on engagement of GT for Black males. The composition of the groups was balanced based on expertise and clinical or lived experience. Two moderators used the same discussion guide that asked participants to discuss factors of the SEM at the individual, interpersonal, community, organizational and policy level that positively or negatively impacted GT for PCA. Discussions were audio recorded and were transcribed by a professional transcription company. Two members of the team (VG and AL) used the moderator guide to develop a codebook for analyzing the transcript from each discussion. Coding utilized an inductive approach [47], to determine key factors for each SEM level. Any discrepancies in coding were discussed until agreement was reached. A full description of key themes identified are in the Supplement.

### Development of consensus statements and consensus process

**Fig. 2:**
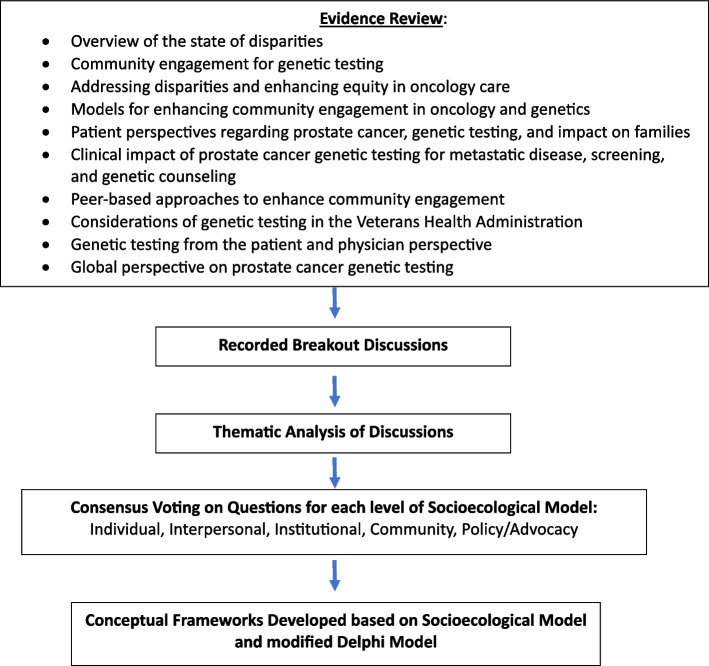
Methods of equity framework development. Stepwise process for development of qualitative and quantitative methods to develop conceptual frameworks for engagement of Black males in prostate cancer genetic testing

Informed by the results of the thematic content analysis and guided by the modified Delphi model [48], a series of statements were developed to address barriers to engagement in PCA GT for Black males at each level of the SEM (69 statements), as well as barriers to and opportunities for engaging Black males in PCA genetics research (27 statements) (Supplemental file). Responses were on a 5-point Likert scale (1 = Strongly Disagree to 5 = Strongly Agree). Votes were cast anonymously using a Web-based survey platform (Qualtrics). Strength of consensus was > = 75% agreement for strong consensus, 50–74% agreement for moderate consensus, and < 50% agreement for lack of consensus. Figure 2 summarizes the methods towards development of the conceptual models.

### Development of equity frameworks

Two conceptual frameworks for increasing engagement of Black males in PCA GT were developed: one for the community/clinical context and a second for the research context based on the SEM. Statements that garnered strong consensus for strong agreement/agreement were included in the models.

### Regulatory considerations

Participants were invited to participate in the conference by the conference co-chairs and were informed about the intent to publish the responses to the consensus approach employed in this conference through email communication as well as verbally at the start of the conference. The conference was held on June 13, 2023, and July 11, 2023 over Zoom. The conference proceedings process did not meet federal definitions of human subjects research as cited in HHS and FDA regulations at 45 CFR 46.102 & 21 CFR 50.3, respectively, and therefore did not fall under IRB purview and did not require IRB review. This process is supported by prior precedent based on previously published consensus papers [48, 49].

## Results

### What strategies should be employed to increase engagement of Black males in prostate cancer genetic testing?

Each statement receiving strong consensus is displayed in Table 2 along with mean scores (MS) for agreement. Several statements to increase participation in GT at the *individual level* were strongly endorsed, and focused on information for individuals regarding the benefits and limitations of GT, hereditary implications, knowledge of family history, and data protections, which can be delivered to the individual from any level of the SEM. Strategies included presenting information in lay language, contextually relevant for Black males, and in an empowering tone for shared decision-making. Furthermore, mistrust needs to be addressed in the delivery of information (MS 4.48-5.00) (Table 2). At *the interpersonal level*, statements receiving strong endorsement included to promote family and friends to support males and provide culturally-tailored communication strategies, develop peer-based approaches to convey genetic information, and train Black role models as community liaisons/health advocates (MS 4.81-5.00). At the *institutional level*, multiple institutions were presented as having a role in increasing Black males’ interest in PCA GT, such as healthcare systems, genetic counseling programs, and institutions of higher education. There was strong consensus for healthcare organizations to invest in genetics programs, diversify clinical teams and train in mistrust, develop culturally-tailored resource materials, conduct culturally-tailored genetic counseling, and diversify the faculty and student body (MS 4.73-5.00) (Table 2). Statements garnering strong support at the *community level* included that community organizations should partner with healthcare organizations to disseminate scientifically-vetted information to the community, integrate genetics information at healthcare events, and launch culturally-related support groups for males with PCA (MS 4.82–4.91) (Table 2). At the *policy and advocacy level*, there was strong consensus for advocacy organizations to collaborate with healthcare organizations, create policies to standardize genetic testing labs’ policies and procedures, develop policies to protect patient/VA/family member benefits, advocate that Medicare recognize genetic counselors as providers, and simplify GT guidelines (MS 4.57-5.00) (Table 2).

**Table 2:** Clinical/Community strategies meeting strong consensus to increase engagement of Black males in prostate cancer genetic testing

	Mean Score
**(a) **Individual level Several key elements of information were endorsed that Black males need to receive from various levels of the socioecological model to increase awareness of prostate cancer genetic testing. Informational elements achieving strong consensus included:	Rated 1 (Strongly disagree) to 5 (Strongly agree)
Education needs to prioritize prostate cancer genetic testing.	4.83
Education about prostate cancer genetic testing needs to be empowering for Black males.	4.83
Information needs to be relevant to males with and without prostate cancer.	4.83
Educational information needs to include potential benefit of genetic testing.	4.83
Education needs to include information about various types of genetic tests and their pros and cons.	4.74
Education needs to emphasize the importance of knowing family history.	4.57
Education about genetic testing needs to address potential identification of hereditary cancer syndromes.	5.00
Education should address the potential to uncover additional cancer risks for men beyond prostate cancer.	5.00
Address familial impact of genetic testing.	4.83
Address role of genetic testing in prostate cancer screening and shared decision-making.	4.83
Discuss genetic data protections.	5.00
Acknowledge history of mistrust in clinical and community settings.	4.48
In clinical setting, also discuss impact of genetic results on therapeutic decision-making for metastatic disease.	4.65
Present information in plain/lay language.	5.00
**(b) **Interpersonal level Strategies to convey the information to the individual from the interpersonal level achieving strong consensus included:	
Family and friends serve as key educators for men regarding prostate cancer genetic testing in the Black community.	4.82
Family and friends may serve key support role for males considering prostate cancer genetic testing.	5.00
Families should be provided with culturally-tailored communication strategies to enhance trust about genetic testing for prostate cancer.	4.82
Peer-based approaches should be employed to enhance relatability to education about genetic testing and overcome mistrust in the Black community.	5.00
Black males should be trained as community liaisons/health advocates to disseminate tailored messages to the Black community.	4.81
Black male role models are needed for public messaging and enhancing trust in genetic testing (ex: sports figures, community leaders, etc.)	5.00
**(c) **Institutional level Multiple types of institutions were presented including healthcare, employment, and education. Statements achieving strong consensus included:	
Healthcare organizations need to value genetic testing and invest in cancer genetics programs.	4.91
There is a need to diversify the workforce of clinicians and genetic counselors so Black males can see themselves in trusted roles.	4.91
Healthcare organizations need to commit resources to hiring more Black male genetic counselors.	4.73
Physicians need formal learning about medical mistrust that Black patients may experience.	4.91
Healthcare providers need to be trained in culturally-competent language to introduce genetic testing to Black males.	4.82
Physicians need readily accessible clinical tools to operationalize guidelines and identify males who may benefit from genetic counseling and genetic testing.	5.00
Healthcare organizations need to commit resources to hiring more community liaisons/health advocates/patient navigators to bridge the gap between healthcare and community regarding prostate cancer genetic testing.	4.91
There is a need to increase the presence of Black male community health workers in healthcare teams to increase relatability and engagement in prostate cancer genetic testing.	4.73
Healthcare organizations need to develop culturally-tailored resource materials and clinical tools to implement prostate cancer genetic testing.	5.00
Culturally-appropriate genetic counseling needs to be conducted to enhance patient relatability to genetic testing.	4.91
Colleges and universities, especially Historically Black Colleges and Universities, should promote genetic counseling as a career.	5.00
Genetic counseling degree programs need diversify student body.	4.82
Genetic counseling degree programs need diversify faculty body.	4.91
**(d) **Community level Community engagement strategies achieving strong consensus included:	
Trusted community organizations should partner with healthcare organizations to provide a link between raising community awareness to clinical care regarding prostate cancer genetic testing for Black males.	4.91
Community health events need to integrate information about prostate cancer genetic testing.	4.82
Culturally-related support groups are needed for Black males with prostate cancer to consider genetic testing.	4.82
**(e)**Policy and advocacy level Multiple strategies were endorsed addressing policy and advocacy impacting all elements of the conceptual framework.	
Advocacy organizations play a key role to raise awareness about prostate cancer genetic testing.	4.83
Greater collaboration is needed between clinicians, genetic counselors, and Black advocacy organizations to reduce disparities in engagement of Black males in prostate cancer genetic testing.	4.83
Policies need to be created to standardize genetic labs regarding testing, payment, and privacy.	4.57
Overall there needs to be more testing of diverse patient populations to help with variant classification.	4.83
Policies are needed to ensure benefits (life insurance) remain regardless of genetic test result.	5.00
Veterans Affairs need to protect the benefits of veterans regardless of genetic test result.	5.00
Policies are needed to protect family members who have genetic testing so benefits are not at risk.	5.00
Medicare needs to recognize genetic counselors as providers to streamline care and reduce barriers.	4.91
There is a need to simplify genetic testing guidelines for greater implementation in clinical practice.	4.57
Media messages Relevant to all levels of the SEM	
Media messages (press, online, radio, TV) regarding prostate cancer genetic testing need to be empowering for Black males.	5.00
Media information (press, online, radio, TV) about prostate cancer genetic testing needs to be culturally-tailored for relatability for Black males.	5.00
Social media campaigns need to be targeted for Black males about prostate cancer genetic testing.	4.57
Culturally-tailored podcasts can increase awareness of prostate cancer genetic testing for Black males.	4.74

Strong consensus entails > = 75% voted strongly agree or agree with the statement

Special consideration was given to media avenues for genetics information. These media messages and strategies are cross-cutting across levels of the SEM model; they may reach directly to the individual or through institutions, community organizations, and advocacy organizations. Strategies achieving strong consensus included that media messages need to have empowering content, messages need to be culturally-tailored and relatable, and targeted social media campaigns and podcasts are needed regarding PCA GT (MS 4.57-5.00) (Table 2).

While there was strong consensus for many strategies, not all statements achieved strong consensus. During the discussion, there was interest in delivering information about the function of the prostate gland and male sexual function along with information about PCA GT which did not achieve strong consensus during voting. In the community setting, discussion of how GT can inform PCA treatment did not achieve strong consensus, but did achieve strong consensus when focused questions were asked about this in the clinical setting. There was not endorsement to introduce GT in the workplace setting or conduct GT in community screening or healthcare events. Also, there was not strong consensus that media messages should acknowledge the history of medical mistrust, but rather this should be addressed by other levels of the SEM.

### Conceptual framework displaying relationships between SEM domains

**Fig. 3:**
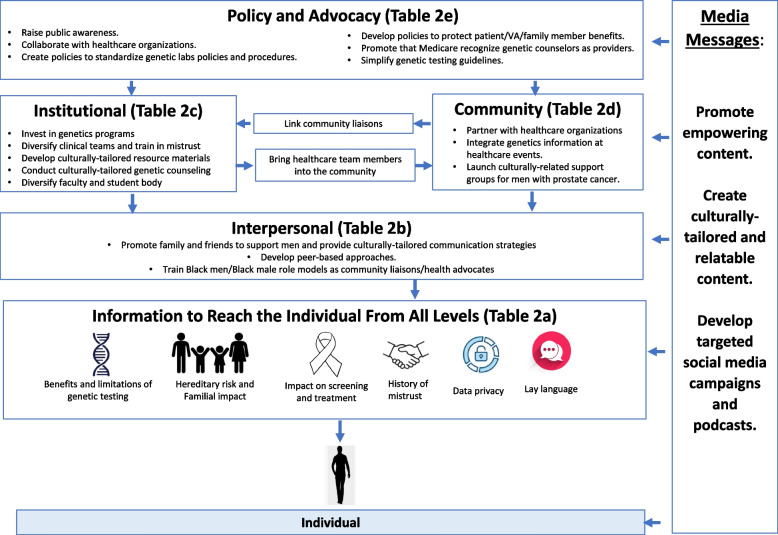
Conceptual equity framework to increase engagement of Black males in prostate cancer genetic testing in community and clinical settings.Elements that achieved strong consensus per each level of the SEM to increase engagement of Black males in prostate cancer genetic testing. Note: Full recommendations along with mean scores for each level are shown in Table 2.

Figure 3 displays the relationships between the domains of the SEM and recommendations from the consensus results. While each domain has unique results, each level can provide key information about PCA GT to individuals through various strategies, such as partnering with community organizations, utilizing media avenues, or developing peer-based approaches to overcome mistrust (Fig. 3).

#### What strategies should be implemented to increase engagement in prostate cancer genetics research?

Multiple statements achieved strong consensus in the various levels of the SEM as shown in Table 3. Strategies at the *individual level* with strong consensus to promote research engagement involved educating Black males about clinical trials, promoting patient agency in research, empowering messages, addressing the history and practices leading to mistrust in research, and discussing data privacy all in lay language (MS 4.65-5.00) (Table 3). Strategies at the *interpersonal level* with strong consensus included to promote family and friends to support males and provide culturally-tailored communication strategies about research, develop peer-based approaches to disseminate research information and garner trust, and train Black role models to discuss research (MS 4.65–4.91) (Table 3). At the *institutional level*, there was strong consensus to diversify research teams, train physicians in research mistrust, develop culturally-tailored resource materials, and foster diversity in applicants for research education and careers (MS 4.57–4.91) (Table 3). At the *community level*, strategies that received strong consensus included to partner community organizations with healthcare organizations to disseminate research information, raise awareness about genetics research, and foster trust (MS 4.65–4.91) (Table 3). Lastly, at the *policy level*, statements achieving strong support included that advocacy organizations raise public awareness about genetics research, increase and facilitate global PCA genetics research, and gather diverse research participant data to drive policy and practice (mean score for all items 4.83) (Table 3).

**Table 3:** Strategies with strong consensus to increase engagement in prostate cancer genetics research

	Mean Score
**(a) **Individual level Several key elements were endorsed that Black males need to receive from various levels of the socioecological model to increase awareness and engagement in prostate cancer genetics research. Informational elements achieving strong consensus included:	Rated 1 (Strongly disagree) to 5 (Strongly agree)
Education about participation in clinical trials and prostate cancer genetics needs to be empowering for individuals in the Black community.	4.65
Education about participation in clinical trials and prostate cancer genetics research needs to acknowledge history of research abuses and pursuant mistrust.	4.91
The benefit of clinical trials and prostate cancer genetics research needs to be a part of education for individuals in the Black community.	4.83
Patient agency for research consent (decision to participate, continued participation, or withdrawal of participation) in clinical trials or prostate cancer genetics research needs to be emphasized during education in the Black community.	4.91
Education about clinical trials and prostate cancer genetics research needs to include transparency of data use.	5.00
**(b) **Interpersonal level Strategies to convey research information and to provide support to the individual from the interpersonal level achieving strong consensus included:	
Family and friends serve as key advocates for Black males regarding engaging in prostate cancer genetics research or clinical trials.	4.65
Families should be provided with culturally-tailored communication strategies to enhance trust about prostate cancer genetics research and clinical trials.	4.65
Peer-based approaches may be useful to deliver education about prostate cancer genetics research and clinical trials and enhance trust.	4.74
Black male role models (ex: athletes, singers, actors, community leaders, etc.) who have had prostate cancer and/or genetic testing are needed for public messaging and enhancing trust in prostate cancer genetics research and clinical trials.	4.91
**(c) **Institutional level The focus was mostly on healthcare organizations and/or academic centers from which research is conducted. Statements achieving strong consensus included:	
There is a need to diversify the workforce of research staff so Black males can see themselves in trusted research roles.	4.74
There is a need to diversify research investigators to include more Black male researchers enhance trust in research.	4.74
Healthcare organizations need to commit resources to hiring more Black male research team members.	4.74
Physicians need more formal learning about research mistrust that Black participants may experience when introducing research studies.	4.83
Healthcare providers need to be trained in culturally-competent language to introduce prostate cancer genetic testing studies or clinical trials to Black males.	4.57
There is a need to increase engagement of Black male researchers and research team members in community settings to increase relatability for prostate cancer genetics research and clinical trials.	4.91
Healthcare organizations need to develop culturally-tailored resource materials (print, online, etc.) to convey information about research studies.	4.74
Institutions of higher education need to foster Black males to enter medical and research careers.	4.91
**(d) **Community level Community engagement strategies achieving strong consensus included:	
Trusted community organizations should partner with healthcare organizations to provide a link between community awareness to engagement in prostate cancer genetics research and clinical trials.	4.91
Community healthcare and cancer screening events should integrate information about the importance of participation in prostate cancer genetics research and clinical trials.	4.65
**(e) **Policy and advocacy level Multiple strategies were endorsed addressing policy and advocacy impacting all elements of the conceptual framework.	
Advocacy organizations can play a key role in raising awareness of the importance of prostate cancer genetics research and clinical trials for Black males.	4.83
Global research efforts should be advocated to identify genetic mutations of prostate cancer risk across populations of African descent.	4.83
Genetic data from diverse patient populations are required to direct policy and allocation of health system resources for genetic and genomic medicine.	4.83
Media messages Relevant to all levels of the SEM	
Media messages (press, online, radio, TV) regarding importance of research participation need to be empowering for Black males.	4.73
Media information (press, online, radio, TV) about the importance of research participation needs to be culturally-tailored for relatability for Black males.	4.91

Strong consensus entails > = 75% voted strongly agree or agree with the statement

Again, special consideration was given to media avenues for delivery of genetics research information. Strategies achieving strong consensus included that media messages need to be empowering for Black males regarding research participation, and that messages need to be culturally-tailored for relatability when conveying information about research participation (MS 4.73–4.91) (Table 3).

Lower/moderate consensus was observed regarding specific aspects of use of media messages for delivery of genetics research information: acknowledging history of mistrust by the media and use of culturally-tailored podcasts to deliver genetic research information.

### Conceptual framework displaying relationships between SEM domains

**Fig. 4:**
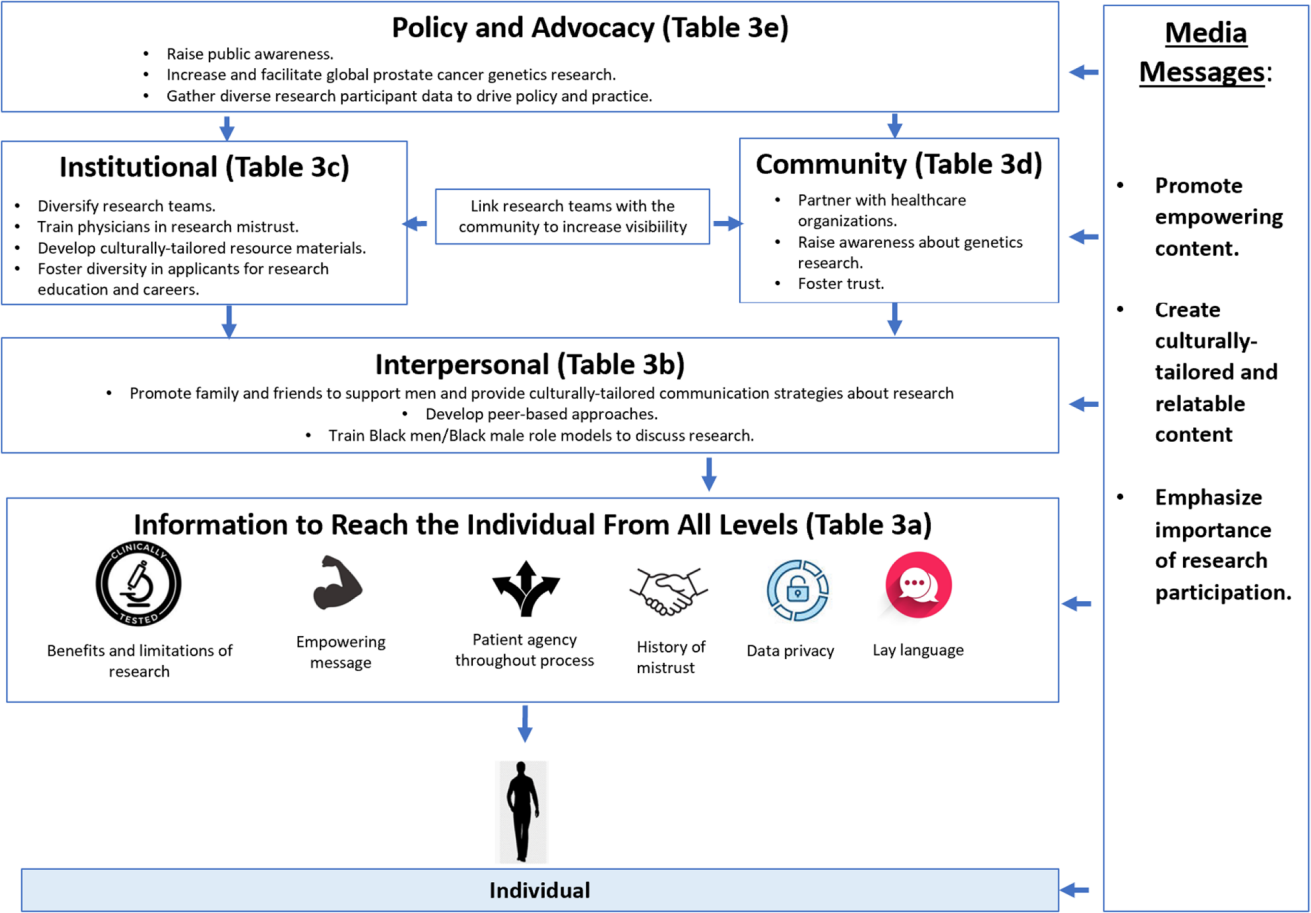
Conceptual equity framework to increase engagement of Black males in prostate cancer genetics research. Elements that achieved strong consensus per each level of the SEM to increase engagement of Black males in prostate cancer genetic testing in the research context. Note: Full recommendations and mean scores for each level are shown in Table 3

Similar to the clinical/community context, the conceptual model in Fig. 4 displays the relationships between the domains of the SEM and recommendations for increasing research engagement from the consensus results. Again, while each domain has unique results, each level can provide key information about PCA genetics research to individuals through various strategies, such as leveraging media avenues, linking healthcare/research organizations with community organizations, or developing peer-based approaches to overcome mistrust (Fig. 4).

## Discussion

The rising role of GT in informing treatment, management, and screening for PCA has heightened the urgency to address disparities [2–5]. While many prior studies have identified barriers that impact cancer disparities, under-engagement in PCA GT remains and is critical to address to ensure equity in PCA care. Implementing strategies for equity in genetics requires consideration of many levels of society to address healthcare systems barriers, cultural beliefs, mistrust of healthcare, and policy bottlenecks that affect engagement of Black males in GT. Thus the SEM was employed to develop comprehensive implementation frameworks for clinical/community engagement and research engagement for PCA GT for Black males. The frameworks are the first to capture multidisciplinary clinical expertise in PCA treatment, cancer, and genetics, community perspectives, and national organization input specifically regarding PCA GT to ensure equity in PCA GT from all levels of society.

The methodology to develop the frameworks was unique in first having open discussion with thematic analysis to garner perspectives which then informed strategies for consensus voting to increase engagement of Black males in PCA GT. The conceptual frameworks for clinical/community and research engagement highlight the interconnectedness of multiple parts of society including interpersonal, institutional, community, and policy/advocacy all of which impact the individual. There are multiple aspects of the results to highlight. A major point during group discussions was that all segments of society (healthcare institutions, medical professional organizations, community members, policy-makers, etc.) need to make awareness of GT a key priority and take responsibility for delivery of this information to Black males from all levels. The information content spans the nature of hereditary GT, benefits and limitations, impact on familial cancer risk, data privacy, and acknowledging mistrust, which remains a major barrier to participating in PCA GT. Interpersonal strategies were therefore viewed as critical to overcome mistrust and have trusted community or family members deliver information to Black males. Here, tools and strategies to support friends and family to facilitate communication about GT are critical [50]. 

Another highlight from the consensus process was the connection between institutional and community domains. While the original SEM depicted community as overarching to institutional levels, our results highlighted the need for strong partnerships between healthcare/educational institutions, community organizations, and advocacy organizations. These results support the development of strategic priorities for healthcare organizations, academic centers, and institutions of higher education to partner with community organizations to raise visibility of researchers and clinicians in the community, and integrate community members in clinical and research teams to achieve the long-term goal of engagement of Black males in PCA GT. Such priorities require funding resources and close engagement of cancer genetics programs with community outreach and engagement teams to disseminate information about PCA GT and develop approaches to overcome barriers to participation in GT.

A unique adaptation of the SEM was the inclusion media messages, which impacted every level of the SEM. Media messages also achieved consensus as impactful strategies to raise awareness of PCA GT and motivate engagement. Media (such as social media, TV, radio) messages can be delivered from institutions, community organizations, advocacy organizations, and individuals/community role models to disseminate information about PCA GT to Black males. Previous studies have shown substantially less social media engagement about *BRCA1/2* and GT in PCA compared with breast cancer [51], highlighting opportunities for institutions, community organizations, advocacy organizations, and peer groups to leverage social media to raise awareness of PCA GT for Black males. Interestingly, while acknowledging mistrust in genetics and healthcare achieved strong consensus as part of the information to deliver from the various levels of the model, this did not achieve strong consensus for integrating into media messages. More insights and research into the use of various media venues to deliver PCA genetics information is warranted.

Multiple strategies for policy and advocacy were endorsed to increase engagement of Black males in PCA GT. The importance of providing GT to diverse populations has become paramount with recognition of higher rates of variants of uncertain significance (VUS) in non-White populations [2–4, 13–15]. As multigene testing has become standard of care, informative genetic test results for Black males are needed to guide PCA treatment, screening, and hereditary cancer management in families, all of which require greater engagement in clinical testing and genetics research. The greater reach of advocacy organizations to raise awareness of GT in the Black community is of key importance. National policies that ensure insurance protections are critically needed, to build greater trust in GT among marginalized populations.

There were some considerations to note. Discussion of the importance of GT in PCA treatment achieved strong consensus only in the clinical setting and not in the community setting. Discussion of prostate function was felt to be of importance during open discussion; however, statements addressing overall prostate function did not achieve strong consensus in the voting session. Nevertheless, there was support to place genetics in the context of overall health and wellness amongst the participants. While there is a strong motivation to make GT more accessible, offering GT at community healthcare events, cancer screening events, or in the workplace was not endorsed given the need for appropriate pretest education and informed consent. Further ways to leverage these intersections of employment and community events with GT may be opportunities to explore with attention to responsible genetics care delivery.

## Conclusions

Adaptation of the SEM using mixed methodology resulted in the first set of equity conceptual frameworks incorporating perspectives from multidisciplinary stakeholders and community members to endorse strategies to increase engagement of Black males in PCA GT – an area of critical need to reduce disparities in PCA outcomes by informing tailored screening, targeted therapy, and cascade testing in families. Individuals, communities, and organizations can utilize the models from their vantage point to raise awareness of PCA GT, build key partnerships, and improve PCA outcomes for Black males. The strategies endorsed here provide practical guidance for implementation across sectors of clinical care, research, and policy and provide groundwork to support equity-driven research in PCA GT.

## Supplementary Information

Supplementary Material 1.

Supplementary Material 2.

## Data Availability

Data are available upon request.
