# Supplementary material for: Adaptation of the socioecological model to address disparities in engagement of Black men in prostate cancer genetic testing
Source: BMC Public Health. 2024 Sep 18;24:2533. doi: 10.1186/s12889-024-20008-8 (PMC11409532; doi:10.1186/s12889-024-20008-8)
Supplement: Supplementary file 1 — Supplementary Material 1. [file 12889_2024_20008_MOESM1_ESM.docx]

**Themes from Breakout Sessions**

**Need to clarify:**

- Cancer patients – addressing barriers at individual, interpersonal, and healthcare organizations
  - Diversify the medical and genetics workforce
  - Consider policy to address financial concerns
  - Greater ways to make GT more feasible in busy practices
  - VA as special HCO
- Noncancer patients – addressing barriers at individual, interpersonal, provider (like PCP) and community perspective.
  - Emphasis in the community of why this is PCA GT is important. Strategies to hammer it home.
  - Information needs to be conveyed in an understandable way – important to have intentionality vs. Default
  - Discussion of genetic testing as a way to better estimate risk for PCA, not a definitive that someone will get cancer.
- General points that affect both cancer patients and non-cancer patients:
  - Cannot create a monolith of what all Black men are the same
  - Trust has to be addressed across both groups.
  - Address sexuality concerns
  - Public communication around different types of tests (DTC vs. clinical testing)
  - Increase online information and communication
  - Package communication so that it is relatable and increase acceptability
  - Educate urologists and PCPs about GT
  - Importance of spouses, daughters, sons, friends to advocate

**Individual Level Barriers:**

--Genetic testing being more associated with women (breast cancer) and families – men not thinking it is for them

--Too many office visits to get genetic testing -. need to integrate it into first urology visit

--Lack of understanding about the prostate and prostate cancer

--Lack of understanding about genetic testing and its purpose/importance; confusion with at-home testing kits and what they do vs. what genetic testing for prostate cancer does

--Concern over the implications (risks?) of genetic testing and the security of the information (company having DNA, insurance being impacted)

--Historical exclusion or abuse in science and research -> lack of trust

--Men being more concerned with the physical effects of prostate cancer (urination, sexual function) and less concerned about genetics and family history. Also, men equating the prostate with sexual function and not wanting to talk about it in a public setting or with others

--The masculinity of men and not wanting to discuss issues of the prostate or health – being a ‘strong Black man’

--Men not wanting to know the results of the genetic test -> would rather “live my life”

--Cost of testing (and any follow-up that might be needed)

**Solutions to Individual Level Barriers:**

--Genetic testing for all men (regardless of race, for an equity approach), at low/no cost

--Personalized care and personalized conversations

--More consistent messaging about genetic testing, especially in places that men will see it (sports events, billboards, commercials)

--Normalizing the conversation about prostate cancer and genetic testing, similar to that with women and breast cancer

--Building relationships and trust takes time -> have a consistent presence (in the clinic, in the community, etc.)

**Interpersonal Level Barriers:**

--Men don’t have conversations with other men about prostate cancer or genetic testing; there’s a stigma in talking about it

--Lack of support in the Black male community to discuss prostate cancer; lack of encouragement to get tested

--Men not wanting to have to share/burden family members (children) with news of a genetic mutation that could impact them

--Lack of representation in clinicians/staff or other patients who are receiving genetic testing -> sends an impression that this “is not for me” or can’t be trusted

**Solutions to Interpersonal Level Barriers:**

--Younger generation family members can be persuasive, and knowledgeable, about health issues impacting older men in their family

--Bringing along a support person to medical appointments is helpful in processing and remembering information

--Network (peer based) approach, having groups of men with similar lived experiences supporting one another through genetic testing

**Organizational Level Barriers:**
--No centralized way to order genetic testing -> need to streamline the process

-Workforce development: more funds and resources to hire genetic coordinators, liaisons that mirror the community and make genetic testing easier

--Guidelines are confusing, primary care and other providers are unclear when to order screening or genetic testing

**Solutions to Organizational Level Barriers:**

--Genetic testing companies should cover the costs of testing for low-income patients

--Changing institutional culture to value genetic testing and expect it for all eligible patients

--Diversify the workforce, so Black men see people like them in trusted roles (clinician, genetic counselor)

**Community-Level Barriers:**

--Lack of media/spokespersons

--Media talks a lot about women and hereditary breast cancer but not men and prostate cancer

**Solutions to Community-Level Barriers:**

--Simplifying the message, to make it easier for men to understand when to test

--Social media campaigns, targeted to Black men

--Media messages should be informative, but also empowering for men to not be afraid to take action

--Offer health workshops in the community and at large events/gathering of men -> bring genetic testing out of the lab/hospital and into the community

--Partner with trusted community organizations (and churches) to host screening and testing events

**Policy-Level Barriers:**

--men are worried about losing health benefits with a positive test result

**Solutions to Policy-Level Barriers:**

--Create policies that mandate insurance companies to cover the cost of testing without out-of-pocket expenses

--Create policies to ensure benefits remain regardless of test result

--Create policies to standardize processes across genetic labs, regarding testing, privacy, assurances, payment, billing

--Guidelines for BRCA2 positive men

--Simplify and streamline the guidelines – current lack of clarity on recommendations

--Policies to protect family members of those who were tested – to ensure that family members don’t lose coverage

--Guidelines to address genetic testing for men with low risk of prostate cancer

--Remove family history criteria from the guidelines -> too many people don’t know their family history
