# Supplementary material for: Adaptation of the socioecological model to address disparities in engagement of Black men in prostate cancer genetic testing
Source: BMC Public Health. 2024 Sep 18;24:2533. doi: 10.1186/s12889-024-20008-8 (PMC11409532; doi:10.1186/s12889-024-20008-8)
Supplement: Supplementary file 2 — Supplementary Material 2. [file 12889_2024_20008_MOESM2_ESM.docx]

| **Clinical and Community Strategies Based on Socioecological Model: Questions for Consensus Voting on a 5-point Likert scale*** |
| --- |
| **Individual Strategies** |
| 1. Education about prostate cancer genetic testing needs to be prioritized for individuals in the Black community. |
| 1. Education about prostate cancer genetic testing needs to be empowering for Black men. |
| 1. Education about prostate cancer genetic testing for individuals in the Black community needs to be inclusive of men with prostate cancer and without prostate cancer. |
| 1. Education about prostate cancer genetic testing for individuals in the Black community needs to include discussion of potential benefit of genetic testing. |
| 1. Education about prostate cancer genetic testing for individuals in the Black community needs to include information about various types of genetic tests and their pros and cons. |
| 1. Education about prostate cancer genetic testing for individuals in the Black community needs to emphasize family history collection (to best that the information is available). |
| 1. Education about prostate cancer genetic testing for individuals in the Black community needs to include discussion of what is the function of the prostate gland. |
| 1. Education about prostate cancer genetic testing for individuals in the Black community needs to include information about male sexual function and distinction from genetic testing. |
| 1. Education about prostate cancer genetic testing for individuals in the Black community needs to address potential identification of hereditary cancer syndromes. |
| 1. Education about prostate cancer genetic testing for individuals in the Black community needs to address the potential to uncover additional cancer risks for men beyond prostate cancer. |
| 1. Education about prostate cancer genetic testing for individuals in the Black community needs to address the familial impact of genetic testing. |
| 1. Education about prostate cancer genetic testing for individuals in the Black community needs to address the connection to informing prostate cancer screening and the need for shared decision-making. |
| 1. Education about prostate cancer genetic testing for individuals in the Black community needs to address potential impact on therapeutic decision-making for metastatic disease. |
| 1. Education about prostate cancer genetic testing for individuals in the Black community needs to address the current state of genetic data protections and limitations. |
| 1. Education about prostate cancer genetic testing for individuals in the Black community needs to acknowledge mistrust in its messaging. |
| 1. Education about prostate cancer genetic testing for individuals in the Black community needs to be in plain/lay language. |
| 1. Education about prostate cancer genetic testing for Black men in the clinical setting needs to include discussion of potential benefit of genetic testing. |
| 1. Education about prostate cancer genetic testing for Black men in the clinical setting needs to include information about various types of genetic tests and their pros and cons. |
| 1. Education about prostate cancer genetic testing for Black men in the clinical setting needs to emphasize family history collection (to best that the information is available). |
| 1. Education about prostate cancer genetic testing for Black men in the clinical setting needs to address potential identification of hereditary cancer syndromes. |
| 1. Education about prostate cancer genetic testing for Black men in the clinical setting needs to address the potential to uncover additional cancer risks for men beyond prostate cancer. |
| 1. Education about prostate cancer genetic testing for Black men in the clinical setting needs to address the familial impact of genetic testing. |
| 1. Education about prostate cancer genetic testing for Black men without prostate cancer in the clinical setting needs to address the connection to informing prostate cancer screening and the need for shared decision-making. |
| 1. Education about prostate cancer genetic testing for Black men with prostate cancer in the clinical setting needs to address potential impact on therapeutic decision-making for metastatic disease. |
| 1. Education about prostate cancer genetic testing for Black men in the clinical setting needs to address the current state of genetic data protections and limitations. |
| 1. Education about prostate cancer genetic testing for Black men in the clinical setting needs to acknowledge mistrust in its messaging. |
| 1. Education about prostate cancer genetic testing for Black men in the clinical setting needs to be in plain/lay language. |
| **Interpersonal Strategies** |
| 1. Family and friends serve as key educators of prostate cancer genetic testing in the Black community. |
| 1. Family and friends may serve key support role for men considering prostate cancer genetic testing. |
| 1. Families should be provided with culturally-tailored communication strategies to enhance trust about genetic testing for prostate cancer. |
| 1. Peer-based approaches should be employed to enhance relatability to education about genetic testing and overcome mistrust in the Black community. |
| 1. Black men should be trained as community liasons/health advocates to disseminate tailored messages to the Black community about prostate cancer genetic testing. |
| 1. Black male role models who have had prostate cancer and genetic testing are needed for public messaging and enhancing trust in genetic testing (ex: sports figures, community leaders, etc.) |
| **Institutional Strategies** |
| 1. Workplace may serve as a key avenue to introduce prostate cancer genetic testing to Black men. |
| 1. Healthcare institutional culture needs to change to value genetic testing and support growth of cancer genetics programs. |
| 1. There is a need to diversify the workforce of clinicians and genetic counselors so Black men can see themselves in trusted roles. |
| 1. Healthcare organizations need to commit resources to hiring more Black male genetic counselors. |
| 1. Physicians need formal learning about medical mistrust that Black patients may experience. |
| 1. Healthcare providers need to be trained in culturally-competent language to introduce genetic testing to Black men. |
| 1. Physicians need readily accessible clinical tools to operationalize guidelines and identify men who may benefit from genetic counseling and testing for prostate cancer. |
| 1. Healthcare organizations need to commit resources to hiring more community liasons/health advocates to bridge the gap between healthcare and community regarding prostate cancer genetic testing. |
| 1. There is a need to increase the presence of Black male community health workers in healthcare teams to increase relatability and engagement in prostate cancer genetic testing. |
| 1. Healthcare organizations need to develop culturally-tailored resource materials (print, online, etc.) to convey information about prostate cancer genetic testing. |
| 1. Culturally-appropriate genetic counseling needs to be conducted to enhance patient relatability to genetic testing. |
| 1. Colleges and universities, especially Historically Black Colleges and Universities, should promote genetic counseling as a career. |
| 1. Genetic counseling degree programs need to assure diversity of the student body. |
| 1. Genetic counseling degree programs need to assure diversity of the faculty body. |
| **Community Strategies** |
| 1. Trusted community organizations should partner with healthcare organizations to provide a link between raising community awareness to clinical care regarding prostate cancer genetic testing for Black men. |
| 1. Community healthcare and cancer screening events should integrate information about prostate cancer genetic testing for Black men. |
| 1. Community healthcare and cancer screening events should conduct prostate cancer genetic testing for Black men. |
| 1. If community healthcare and cancer screening events conduct prostate cancer genetic testing for Black men, they should be connected with clinics for follow up recommendations and care. |
| 1. Partnerships should be developed between trusted community organizations and genetic counseling programs to host screening and genetic testing events. |
| 1. Culturally-related support groups need to be developed to support Black men with prostate cancer to engage in genetic testing. |
| 1. Media messages (press, online, radio, TV) regarding prostate cancer genetic testing need to be empowering for Black men. |
| 1. Media information (press, online, radio, TV) about prostate cancer genetic testing needs to be culturally-tailored for relatability for Black men. |
| 1. Media messages about genetic testing need to acknowledge the history leading to mistrust of genetic testing in the Black community. |
| 1. Social media campaigns need to be targeted for Black men about prostate cancer genetic testing. |
| 1. Culturally-tailored podcasts can increase awareness of prostate cancer genetic testing for Black men. |
| **Policy and Advocacy** |
| 1. Advocacy organizations can play a key role in raising awareness of prostate cancer genetic testing for Black men. |
| 1. Greater collaboration is needed between oncologists, urologists, primary care physicians, and genetic counselors to reduce disparities in engagement of prostate cancer genetic testing. |
| 1. Greater collaboration is needed between clinicians, genetic counselors, and Black advocacy organizations to reduce disparities in engagement of Black men in prostate cancer genetic testing. |
| 1. Genetic testing companies should cover the costs of genetic testing for low-income patients. |
| 1. Policies need to be created to standardize genetic labs regarding testing, payment, and privacy. |
| 1. Diversity in variant detection and curation is needed to ensure we can accurately classify pathogenic variants in all populations. |
| 1. Policies are needed to ensure benefits (healthcare, insurance) remain regardless of genetic test result. |
| 1. The VA needs to protect veterans’ benefits regardless of genetic test result. |
| 1. Policies are needed to protect family members who have genetic testing so benefits are not at risk. |
| 1. Medicare needs to recognize genetic counselors as healthcare providers to enhance streamlined genetic testing operations. |
| 1. Genetic testing guidelines need to be simplified to facilitate identification of men for prostate cancer genetic testing. |

1=Strongly Disagree to 5=Strongly Agree

| **Research Engagement Strategies Based on Socioecological Model: Questions for Consensus Voting on a 5-point Likert scale*** |
| --- |
| **Individual Strategies** |
| 1. Education about the importance of participation in clinical trials and prostate cancer genetics research needs to be empowering for individuals in the Black community. |
|  |
| 1. Education about participation in clinical trials and prostate cancer genetics research needs to acknowledge the history of research abuses and pursuant mistrust in its messaging. |
| 1. The benefit of clinical trials and prostate cancer genetics research (direct benefit to participants, benefit to society, etc) needs to be part of education for individuals in the Black community. |
| 1. Patient agency for research consent (decision to participate, to continue to participate, or withdraw participation) in clinical trials or prostate cancer genetics research needs to be emphasized during education for individuals in the Black community. |
| 1. Education about clinical trials and prostate cancer genetics research needs to include transparency about use of data for individuals in the Black community. |
| **Interpersonal Strategies** |
| 1. Family and friends serve as key advocates for Black men regarding engaging in prostate cancer genetics research or clinical trials. |
| 1. Families should be provided with culturally-tailored communication strategies to enhance trust about prostate cancer genetics research or clinical trials. |
| 1. Peer-based approaches may be useful to deliver education about prostate cancer genetics research and clinical trials and enhance trust for individuals in the Black community. |
| 1. Black male role models who have had prostate cancer and/or genetic testing are needed for public messaging and enhancing trust in prostate cancer genetics research and clinical trials (ex: sports figures, community leaders, etc.) |
| **Institutional Strategies** |
| 1. There is a need to diversify the workforce of research staff so Black men can see themselves in trusted research roles. |
| 1. There is a need to diversify the research investigators to include more Black male researchers so Black men can see themselves in trusted research roles. |
| 1. Healthcare organizations need to commit resources to hiring more Black male research team members. |
| 1. Physicians need formal learning about research mistrust that Black patients may experience when introducing research studies. |
| 1. Healthcare providers need to be trained in culturally-competent language to introduce prostate cancer genetics studies or clinical trials to Black men. |
| 1. There is a need to increase engagement of Black male researchers and research team members in community settings to increase relatability and engagement prostate cancer genetics research and clinical trials. |
| 1. Healthcare organizations need to develop culturally-tailored resource materials (print, online, etc.) to convey information about research studies. |
| 1. Institutions of higher education need to foster Black men to enter medical and research careers. |
| **Community strategies** |
| 1. Trusted community organizations should partner with healthcare organizations to provide a link between community awareness to engagement in prostate cancer genetics research and clinical trials for Black men. |
| 1. Community healthcare and cancer screening events should integrate information about importance of participation in prostate cancer genetics research and clinical trials for Black men. |
| 1. Media messages (press, online, radio, TV) regarding importance of research participation need to be empowering for Black men. |
| 1. Media information (press, online, radio, TV) about the importance of research participation needs to be culturally-tailored for relatability for Black men. |
| 1. Media messages about research participation need to acknowledge the history leading to mistrust in the Black community. |
| 1. Social media campaigns need to be targeted for Black men about importance of participating in research. |
| 1. Culturally-tailored podcasts can increase awareness of research participation for Black men. |
| **Policy and Advocacy** |
| 1. Advocacy organizations can play a key role in raising awareness of importance of prostate cancer genetics research and clinical trials for Black men. |
| 1. Global research efforts should be advocated to identify genetic mutations of prostate cancer risk across populations of African descent. |
| 1. Genetic data from diverse patient populations are required to direct policy and allocation of health system resources for genetic and genomic medicine. |

1=Strongly Disagree to 5=Strongly Agree
